# How to Prevent Mammary Abscess

**Published:** 1879-10

**Authors:** 


How to Prevent Mammary Abscess.—Dr. Francis J. Shepherd, M. R. C. S., Eng., in the *Maryland Medical Journal*, gives the "old wife's remedy," which he has employed with uniform success. "Take a large piece of sticking plaster and cut it a circular shape; make a hole large enough for the nipple and half of the areola to be seen, and apply this plaster, after heating, so that it will cover the whole breast and the nipple will protrude through tho aperture." The plaster should be re-applied when the breast softens and subsides, which will occur generally in twenty-four hours. The late Dr.

Whitehead, of Vicksburg, Miss., was the first to call attention, through the medical press, to the value of strapping the breast as a means of absorbing mammary abscess. The writer had frequently adopted this plan before Dr. Whitehead's publication, and he still uses it with great confidence in its efficacy. It is not well to wait until there are marked evidences of inflammation. A good rule is to ask the patient if the breast feels heavy and drag so much to the side as to impede the movements of her arms, and particularly to prevent her from bringing her arms close to the side of the body. This being the case, take two or three broad strips of plaster (which if good will not need heating), direct the nurse to lift the breasts gently toward the median line of the body upon the sternum, then apply one strip on the lower half of the breast, the other on the upper half, reaching from one axilla to the other. Other auxilliary strips may be applied according to the size of the breasts and effectiveness of the first two. When the breasts have become "caked," and the patient is so uncomfortable as to be unable to sleep, this procedure will generally be followed by immediate relief from discomfort, and the milk will oftentimes flow from the nipple in streams. We have not been successful with the "old wife's remedy" as described by Dr. S.

